# Comparative Analysis of the Tribological Properties of Hexadecanol and Hexadecanethiol as Lubricating Additives in Synthetic Oils

**DOI:** 10.3390/ma19143085

**Published:** 2026-07-17

**Authors:** Chaoyang Zhang, Junyu Fan, Leilei Li, Chuxiang Zhou, Gang Wu, Shuai Hu, Mohamed Kamal Ahmed Ali

**Affiliations:** 1 Sustainable Energy and Environment Thrust and Guangzhou Municipal Key Laboratory of Materials Informatics, The Hong Kong University of Science and Technology (Guangzhou), Guangzhou 511400, Chinajfan822@connect.hkust-gz.edu.cn (J.F.); 2 School of Civil Engineering, Northwest Minzu University, Lanzhou 730030, China; 3 CCDC Drilling & Production Technology Research Institute, Guanghan 618399, China; 4 State Key Laboratory of Solid Lubrication, Lanzhou Institute of Chemical Physics, Chinese Academy of Sciences, Lanzhou 730000, China; 5 Automotive and Tractors Engineering Department, Faculty of Engineering, Minia University, El-Minia 61519, Egypt

**Keywords:** lubricant additives, lubrication mechanisms, friction, wear, adsorption simulation

## Abstract

The improved tribological performance of the lubricating oils addresses durability challenges in mechanical systems. Herein, the physicochemical and tribological properties of 1-hexadecanol (16O) and 1-hexadecanethiol (16S) as oil additives for lubricating steel tribosystems were studied under various concentrations. The tribological behavior of lubricated steel surfaces was evaluated using an SRV-IV tribometer. In addition, a simulation was conducted that highlights the mechanism of additive adsorption on metal surfaces based on density functional theory (DFT). The tribological findings demonstrated that the 16O and 16S additives reduced the friction coefficient by 50–52% and wear volume by 79.9–82.6% compared with PAO10 oil. Notably, the 16O additive offers more consistent and concentration-independent friction reduction, while the 16S additive provides higher anti-wear protection at 0.5 wt% concentration due to the formation of a robust Fe-S tribofilm, which results from its stable physical adsorption mechanism based on simulation results. Briefly, this comparative analysis highlights the importance of optimizing additive concentration based on the specific balance of friction and wear requirements in mechanical applications.

## 1. Introduction

Friction and wear in mechanical systems are important causes of energy waste and component failure. Friction consumes about 23% of the world’s energy, and durability problems directly cause more than half of mechanical failures [1]. Lubricating oil, as the “blood” of mechanical equipment, reduces friction and inhibits extreme wear by forming an isolating tribofilm on the rubbing surfaces. Therefore, the development of lubricants is one of the goals of environmental sustainability to conserve energy and material resources and reduce mechanical equipment maintenance expenses [2,3]. Among all kinds of base oils, poly-α-olefin (PAO) synthetic oil is favored because of its excellent viscosity-temperature characteristics, strong oxidation resistance, and low volatilization loss. In particular, PAO 10 with medium viscosity is widely used in high-end equipment such as industrial gears, compressors, and aero-engines [4]. However, the PAO molecule is a pure hydrocarbon chain structure and lacks polar groups that can have a strong effect on the metal surface. It is difficult to form a strong protective tribofilm under boundary lubrication conditions such as low speed and heavy load [5]. It is easy to cause direct contact of metal, a sudden rise in friction, and aggravation of wear [6,7,8]. Therefore, adding a friction modifier with a polar “anchoring head” to PAO 10 has become a common means to improve its boundary lubrication performance [9].

Long-chain fatty alcohol is a kind of friction modifier, which has been studied earlier. Zhang and Hu et al. [10] synthesized melamine long-chain alcohol esters and studied their lubrication performance under a temperature range of 100 °C to 200 °C. Their results showed that by adding it to PAO 4, the wear rate can still be reduced by more than 92% at 200 °C. Moreover, XPS analysis confirmed that the alcohol ester molecules were firmly adsorbed on the metal surfaces through tribochemical reactions. Reddyhoff et al. [11] found that after 1-dodecanol is mixed with hydrocarbon oil, the alcohol molecules will undergo a pressure-induced phase transition in the high-pressure contact area to form a layered solid phase, which can increase the oil film thickness and reduce the viscous friction simultaneously, breaking the traditional understanding that the larger the film thickness, the greater the friction. Simič and Kalin [12] studied the adsorption behavior of hexadecanol on diamond-like carbon (DLC) coatings and steel surfaces using atomic force microscopy and tribological tests. It was found that alcohol molecules can be physically and chemically adsorbed on the DLC surfaces, effectively reducing the wear of the coating, but the reduction in the friction coefficient is limited because the DLC coating itself has lower friction characteristics. In terms of thiols, Marx et al. [13] studied the self-assembled monolayers of hexadecanethiol on a gold surface with atomic force microscopy and found that there are two different mechanisms for the change in friction energy dissipation with temperature and sliding speed. Sano et al. [14] directly compared the SAMs of hexadecanethiol and hexadecanol on silicon substrates. XPS and AFM results showed that the molecular arrangement of the thiol film was not as dense as that of the alcohol film, and the durability in hydrofluoric acid solution was also worse. Xia et al. [15] introduced dodecanethiol (DDT) as a friction modifier and extreme pressure agent with graphene oxide (GO) nanoadditive. Their results demonstrated the good dispersed stability of the GO/DDT additives within the white oil for a long period of up to one year. At the addition of 0.06 wt%, the friction coefficient decreased by 31.9% and the wear scar diameter decreased by 28.8%. The excellent performance was attributed to the physical protective film and chemical reaction film formed on the friction surface by DDT and GO synergistically. Furthermore, the structure of the base oil significantly influences additive performance, as branched fatty acids, while effective in straight-chain hexadecane, show limited effect in branched PAO [16].

Despite these advances, two critical gaps remain. First, most studies investigate alcohols and thiols separately under disparate conditions or compare them only as self-assembled nanoscale films [17,18,19]. Second, a direct, systematic comparison of hexadecanol (–OH) and hexadecanethiol (–SH) with identical carbon chain length (C16) in the same PAO 10 base oil under identical testing conditions is still lacking. In particular, whether thiols truly outperform alcohols due to the formation of iron sulfide tribofilms remains unclear due to insufficient experimental evidence [20,21,22]. Therefore, in this work, hexadecanol (16O) and hexadecanethiol (16S) additives were selected as model friction modifiers, and the tribological properties of the two additives at different concentrations were systematically investigated in PAO 10 base oil. This investigation aims to uncover their lubrication mechanisms under sliding contact conditions.

## 2. Materials and Methods

### 2.1. Materials

In this investigation, the baseline oil for the studied additives was synthetic poly-α-olefin (PAO10). The PetroChina Lanzhou Lubricant Research and Development Center (Lanzhou, China) provided PAO 10 basic oil. Regarding the studied oil additives, 1-hexadecanol (referred to as 16O, purity of 98%, formula: C_16_H_34_O) and 1-hexadecanethiol (referred to as 16S, purity of 97%, formula: C_16_H_34_S) were purchased from Tishai (Shanghai) Chemical Industry Development Co., Ltd. (Shanghai, China).

### 2.2. Lubricant Formulation and Characterizations

The lubricant specimens were prepared by adding hexadecanol (16O) and hexadecanethiol (16S) additives to PAO10 oil at 0.3, 0.5, and 1 wt%. These concentrations were chosen in accordance with the optimal range for organic oil additives, which is normally defined between 0.25 and 2.0% as friction-reducing, which is adequate to reach the FZG 12 load stage [23]. In the lubricant preparation process, the hexadecanol and hexadecanethiol additives were incorporated with PAO10 oil and stirred for 3 h at room temperature. The lube oil samples showed dispersion stability during the experimental period exceeding 5 weeks. The studied oil samples containing hexadecanol were designated as 0.3–16O, 0.5–16O, and 1–16O, while hexadecanethiol was designated as 0.3–16S, 0.5–16S, and 1–16S in the Section 3. Regarding the physicochemical tests, the thermal stability of the studied lubricants was measured by a NETZSCH STA 449 F3 synchronous thermal analyzer (NETZSCH Group, Selb, Germany), and the temperature was increased from 25 °C to 600 °C at a heating rate of 10 °C/min in an N_2_ atmosphere. The kinematic viscosity at 40 °C and 100 °C was measured using a petroleum product kinematic viscometer (SYP1003-III) (Shanghai Yangde Petroleum Instrument Manufacturing Co., Ltd., Shanghai, China), and then the corresponding viscosity indices were calculated. To ensure test reproducibility, these experiments were carried out three times.

### 2.3. Tribology Performance Testing

The tribological properties of the tested additives (16S and 16O) were tested by the SRV-IV friction and wear tester (Optimol Grease Company, Mönchengladbach, Germany) in a ball-on-disk setup as a tribosystem. The rubbing samples (ball and disk) in this investigation were made of AISI 52100 steel materials. A 10 mm diameter ball served as the upper test specimen, while a 24 mm diameter, 7.9 mm thick disk was used as the lower counterpart. The average initial surface roughness (Ra) of the steel disk and the steel ball was 0.02 μm. The friction experimental conditions were a temperature of 25 °C, a load of 200 N, a sliding frequency of 25 Hz, a sliding amplitude of 1 mm, and a test time of 30 min. The amount of lubricant used in each test was 0.3 mL. The chosen applied load and sliding frequency values led to a boundary lubrication regime, as demonstrated by the friction coefficient values discussed in Section 3. The boundary lubrication regime involves the most severe sliding conditions that are necessary to reduce friction and wear. The tests were conducted twice using the same sliding parameters to ensure reliability. The average values are reported with the standard deviation, which represents the experimental error, in Section 3. The friction specimens were cleaned with acetone and dried using hot air after the frictional testing. After the friction experiment, the wear volume of the wear scar on the surface of the steel disc was measured by a non-contact three-dimensional surface profiler (MicroXAM-800, KLA Corporation, Milpitas, CA, USA). The wear volume was quantified via numerical integration in the MicroXAM Analysis software operating in white-light interferometry mode with a 50× Mirau objective. The wear patterns of the wear scar surface were observed by scanning electron microscopy (SEM, JSM-5600LV, JEOL Ltd., Tokyo, Japan). The chemical element state of tribofilm formed on the wear scar surface was analyzed by X-ray photoelectron spectroscopy (XPS, PHI-5702, Physical Electronics, Chanhassen, MN, USA).

### 2.4. Simulation Approach

According to the density functional theory (DFT), the geometry optimization function of the DMol3 module was utilized in the present study, with the generalized gradient approximation (GGA-PBE) selected as the exchange-correlation functional to optimize the structures of the investigated additives. The quality parameter was set to optimize. Calculations were conducted on the optimized models utilizing the energy function. The analysis encompassed the HOMO, LUMO, and quantum chemical characteristics of the evaluated additions.

**Figure 1:**
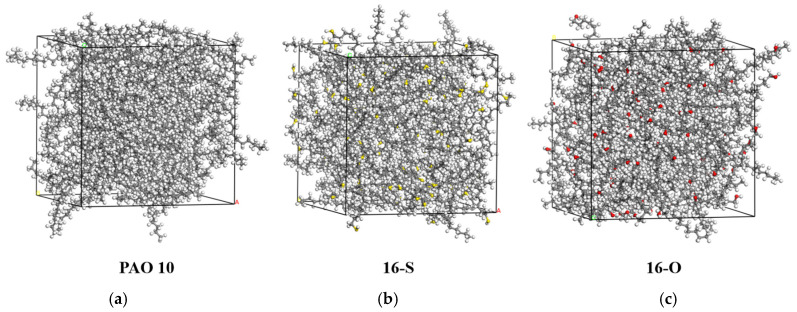
The amorphous unit cell model of the lubrication system with PAO 10 (**a**), 16S (**b**), and 16O (**c**) after structure optimization.

The COMPASS II force field was used to establish an amorphous cell containing PAO10, hexadecanethiol (16-S), and hexadecanol (16-O) using the construction function of the amorphous cell module, where the quality was set to fine and the output was set to 20 frames. The lubrication system with the lowest energy is selected for two geometric optimizations to ensure that the energy of the lubrication system is minimized until convergence is achieved. The optimized composite lubrication system is shown in Figure 1.

The Fe unit cell was imported into the software database, and the Fe (001) surface was selected as the adsorption surface. The thickness was set to 3 layers. A vacuum layer of 30 Å was introduced on the Fe (001) crystal plane, and the unit cell was extended to a 14 × 14 supercell. The optimized PAO10, 16S, and 16O systems were introduced into the established supercell model, and the configuration was used as the adsorption model. The COMPASS II force field was employed in the Forcite module to simulate the adsorption process for 100 ps in the NVT ensemble, with a time step of 1.0 fs. The adsorption energies of adsorbed molecules on Fe surfaces can be calculated according to Equation (1):

(1) $$E_{\mathrm{ads}}=E_{total}-\left(E_{molecule}-E_{Fe(001)}\right)$$

where $E_{\mathrm{ads}}$ represents the adsorption energy of PAO10, 16-S, and 16-O with the Fe (001) surface, kcal/mol; $E_{total}$ is the sum of the energies of PAO10, 16-S, 16-O, and the Fe (001) surface; $E_{molecule}$ represents the energies of PAO10, 16-S, and 16-O, (kcal/mol); and $E_{Fe(001)}$ represents the energy of Fe (kcal/mol).

## 3. Results and Discussion

### 3.1. Lubricant Properties

**Figure 2:**
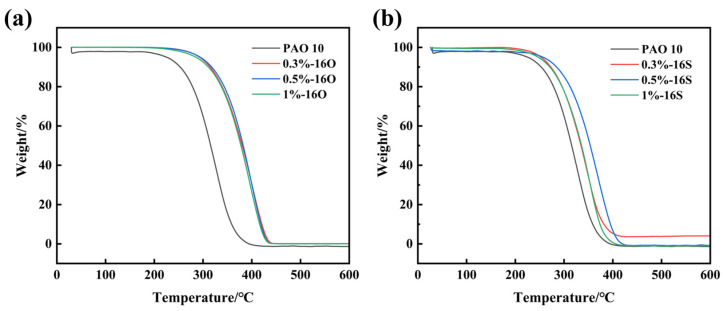
Thermogravimetric curves of investigated lubricants containing 16O (**a**) and 16S (**b**) additives.

The lubricant characteristics are significant as they directly affect the lubrication performance and longevity of lubricated tribopairs [24]. The thermogravimetric curves of the blank sample PAO10 and the prepared lube oils containing 16O and 16S additions with various concentrations are shown in Figure 2. The incorporation of 16O and 16S additives markedly improves the thermal stability of the PAO10 base oil, as illustrated in Figure 2a,b. The pure PAO10 oil has a rapid breakdown beginning at around 250–280 °C, but the samples with 16O and 16S additions showed a notable shift in the TGA curves to elevated temperatures (300–350 °C). This enhancement indicates that the lengthy alkyl chains (C16) of the 16O and 16S additives engage efficiently with the base oil, inhibiting the volatilization and heat degradation of the hydrocarbon chains [25]. Notably, the results revealed that the addition of 16O was superior in its resistance to thermal degradation compared to 16S. The improvement is due to the fact that the carbon-oxygen (C-O) bonds in alcohols are thermally stronger than the carbon-sulfur (C-S) bonds in thiols [26], presenting a lubricant containing a 16O additive as more thermally stable and less prone to premature oxidation compared to a lubricant containing a 16S additive. Furthermore, Figure 2b indicates that the lube oil with a 0.3 wt% 16S addition leaves a measurable solid residue. This note can be linked to the chemical properties of sulfur-containing additives, which are known to decompose to produce metal sulfides at high temperatures [27].

The corresponding thermal decomposition temperatures are shown in Table 1. It can be seen from these results that the thermal decomposition temperature of PAO10 can be increased by adding different mass fractions of 16O and 16S, and the thermal decomposition temperature is the highest at 0.5 wt% additives. The thermal decomposition temperatures of the two friction modifiers at a mass loss of 10% are higher than 270 °C, indicating that they have excellent thermal stability. Hence, the 16O addition at 0.5 wt% is the best choice for practical use in PAO10 oil due to its higher thermal stability. Additionally, it can be seen from the viscosity data in Table 1 that the change in kinematic viscosity of PAO10 at 40 °C and 100 °C is not obvious after adding 16O and 16S, indicating that 16O and 16S have little effect on the viscosity-temperature performance of base oil PAO 10. It is noteworthy that oils containing 16S at the highest concentration showed the highest viscosity index value, reaching 160, despite their thermal stability compared to 16O addition.

### 3.2. Tribological Properties

**Figure 3:**
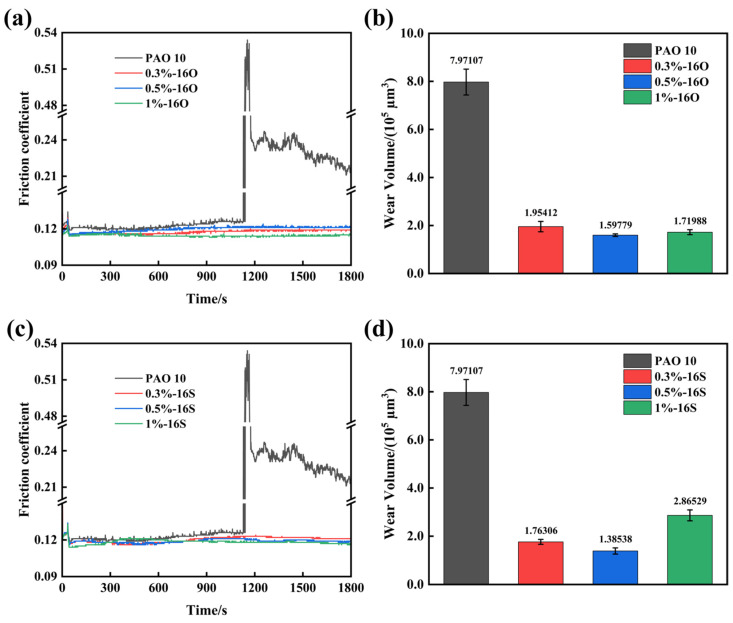
Friction coefficient behavior and wear volume value of disk oiled by lubricants containing 16O (**a**,**b**) and 16S (**c**,**d**) additives with different concentrations.

Figure 3 shows the friction coefficient behaviors and wear volume values measured for the disk lubricated with the tested additives (16O and 16S) under various concentrations. Typically, the coefficient of friction for boundary lubrication ranges from 0.05 to 0.15 [28]. From Figure 3a,c, the PAO10 oil illustrates unstable friction behavior, characterized by a sharp spike in COF (reaching ~0.54) at approximately 1200 s, indicating boundary lubrication failure and direct asperity contact. In contrast, all the tested oils containing the 16O additive maintained a stable and low COF (~0.11–0.12) during the test duration; it is shown that the test is mainly carried out under boundary lubrication conditions. The results showed that the lube oils with various wt% 16O and 16S additives achieved a friction reduction of ~50–52% compared to the unstable baseline of PAO10 oil. This reduction (~50–52%) exceeds the 40–50% reduction reported for long-chain alcohols in PAO [10] and the 31.9% reduction for dodecanethiol in white oil [16], with the COF of ~0.11–0.12 falling within the lower range of reported values (0.10–0.15) for similar C16 additives [13]. In general, the long C16 alkyl chains form a dense, ordered monolayer. This creates a steric barrier that prevents direct metal-to-metal contact, thus reducing friction and adhesive wear [26]. As shown in Figure 3b,d, the results highlighted the higher anti-wear properties of both additives. Remarkably, the lubricant with 16S and 16O at 0.5 wt% exhibited the best overall anti-wear performance, reducing the wear volume by 79.9–82.6% compared with the PAO10 oil. This reduction aligns with the 92% wear reduction reported for melamine alcohol esters in PAO4 oil [10] and significantly surpasses the approximately 28.8% reduction in wear scar diameter observed for GO/DDT in white oil [15].

The tribological improvement provided by the 16O additive is primarily influenced by physical adsorption and hydrogen bonding. The hydroxyl (-OH) group serves as a polar head that effectively adsorbs onto the metal oxide layer (Fe_2_O_3_/Fe_3_O_4_) on the steel interfaces through hydrogen bonding and dipole interactions [29]. Conversely, the superior anti-wear performance of 16S (at 0.5%) is because of the thiol (-SH) group, which undergoes dissociative chemisorption on the iron surface, resulting in the formation of robust Fe-S covalent bonds [27]. Consequently, the tribolayer formed is highly stable and exhibits greater resistance to mechanical shear compared with the physically adsorbed 16O tribolayer. This is consistent with Sano et al. [14], who found that thiols bind more strongly to surfaces than alcohols, though their nanoscale study reported inferior film density for thiols. Our macroscale results in PAO 10 show that stronger chemisorption translates into superior wear protection, suggesting that performance ranking is scale- and condition-dependent. Interestingly, raising the 16S concentration to 1% caused a considerable reduction in wear performance (wear volume increased to 2.86 × 10^5^ μm^3^), but 16O maintained rather steady performance at 1%. High S concentrations can produce corrosive wear, in which the strong reaction with the metal rubbing surface causes pitting and material loss rather than protection.

**Figure 4:**
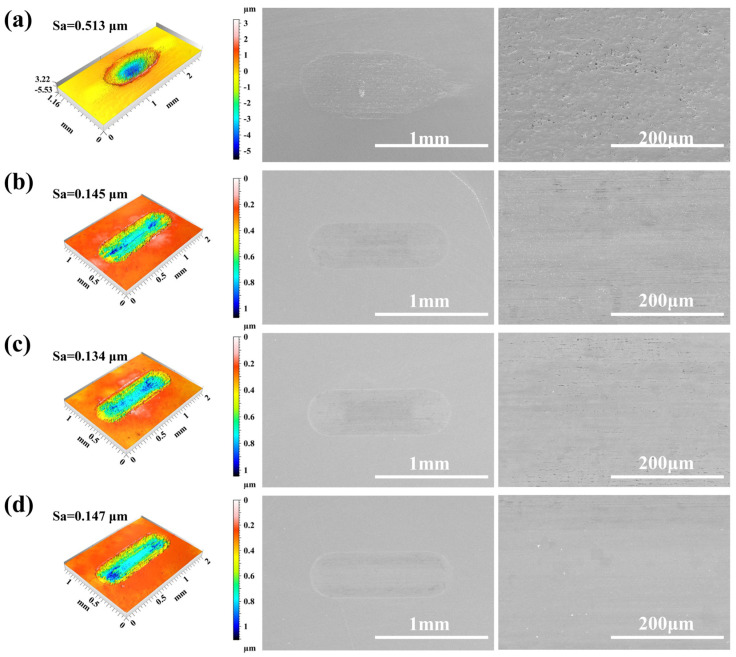
The three-dimensional morphology and SEM images of the tested lubricants containing various 16O concentrations: (**a**) PAO10, (**b**) 0.3 wt% 16O, (**c**) 0.5 wt% 16O, (**d**) 1 wt% 16O.

**Figure 5:**
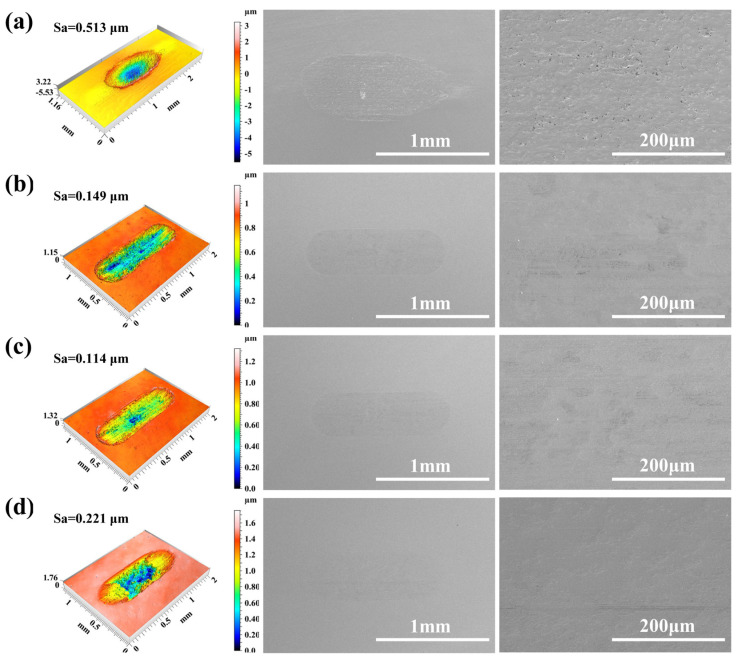
The three-dimensional morphology and SEM images of the tested lubricants containing various 16S concentrations: (**a**) PAO10, (**b**) 0.3 wt% 16S, (**c**) 0.5 wt% 16S, (**d**) 1 wt% 16S.

To display the wear modes, Figure 4 presents the three-dimensional surface topography and SEM micrographs of wear scars oiled with PAO10 and various concentrations of 16O additive (0.3 wt% 16O, 0.5 wt% 16O and 1 wt% 16O). It can be seen from the results that the wear of PAO10 is serious; the wear scar is obviously wider, and the edge is irregular. The three-dimensional topography can be observed to have a large depth, and the obvious adhesive wear, abrasive wear and metal surface peeling can be observed. The SEM images show that the wear scar generated by pure PAO10 exhibits rough, damaged surfaces with discernible grooves and adhesive wear characteristics, while the surfaces lubricated with 16O additions display a notably smooth and uniform morphology with minimal surface degradation. This surface protection is attributed to the formation of an adsorbed boundary film, where the polar hydroxyl (-OH) groups of 16O molecules strongly adsorb onto the metal oxide surface through tribochemical interactions, creating a protective tribolayer. In addition, Figure 5 shows the three-dimensional morphology and SEM image of the disc wear scar lubricated by oils containing 16S additive with varying wt%. It can be seen from the SEM images that the surface of the wear scar oiled by 0.5 wt% 16S is the smoothest, while the wear scar oiled with 1wt% 16S is seriously corroded and worn. It can be seen from the three-dimensional morphology that the depth of the middle area is deeper. When comparing the performance of both 16O and 16S additives, the morphological behavior of the worn surfaces was fairly similar.

**Figure 6:**
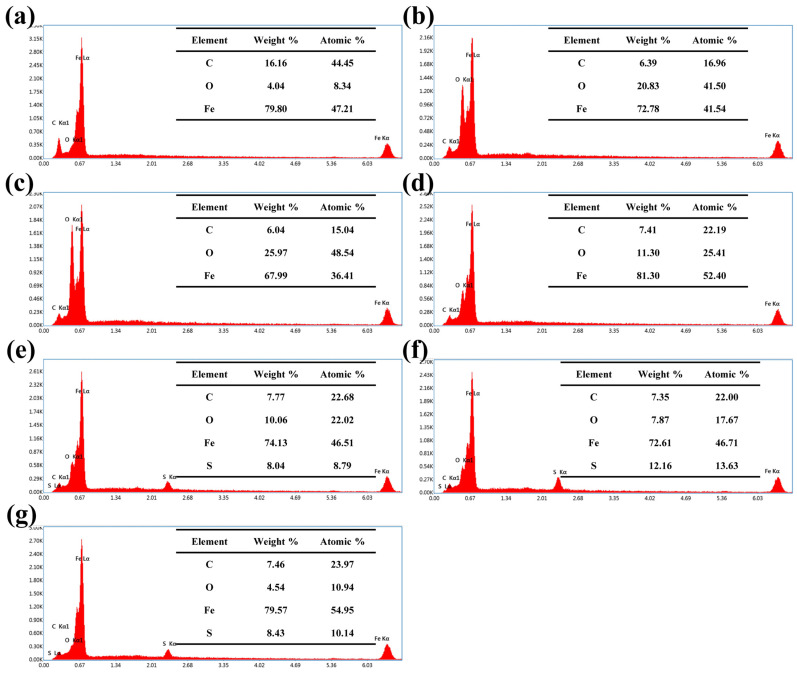
EDS analysis of entire disk wear scar after lubrication by tested oils: (**a**) PAO10, (**b**) 0.3 wt% 16O, (**c**) 0.5 wt% 16O, (**d**) 1 wt% 16O, (**e**) 0.3 wt% 16S, (**f**) 0.5 wt%16S, (**g**) 1 wt% 16S.

Figure 6 shows the EDS analysis results of the wear scar surface after the friction experiment for the tested lubricants. From the data in this analysis, it can be seen that the wear scar surface lubricated by the two friction modifiers can detect its own characteristic elements. The O element of the wear scar surface after 16O lubrication is significantly higher than that of the wear scar surface after PAO10 lubrication, and the S element is also detected on the wear scar surface after 16S lubrication. The contents of characteristic elements O and S detected by 0.5 wt% 16O and 0.5 wt% 16S are also the highest, indicating that the tribochemical reaction occurs with the steel friction pair during the friction process, and the tribochemical reaction film is formed on the worn interfaces, which effectively improves the anti-wear performance of PAO10 oil. This also confirms the conclusion drawn from the friction experiment: adding 0.5 wt% of the tested friction modifier, the anti-wear performance is optimal.

**Figure 7:**
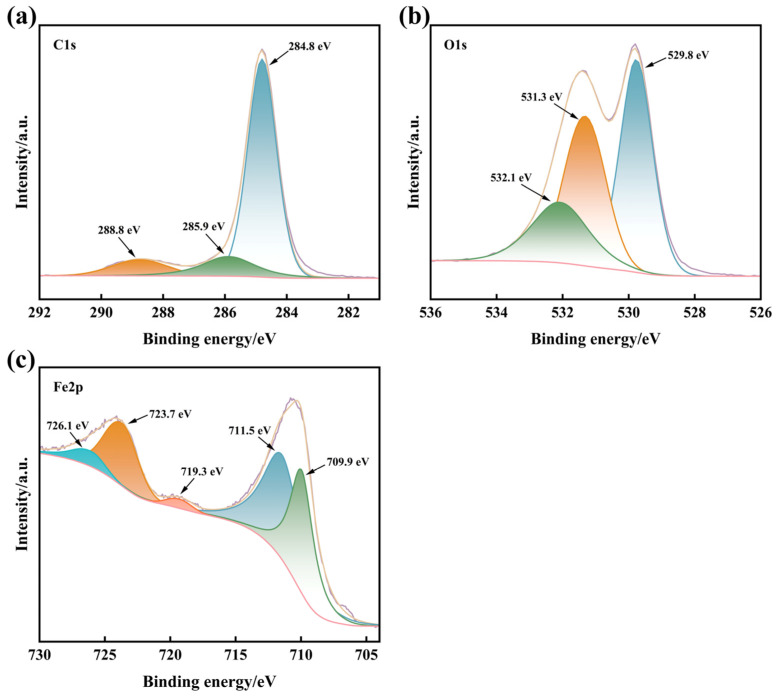
XPS analysis of wear scar surface after 1 wt%16O lubrication: (**a**) C1s, (**b**) O1s, (**c**) Fe2p.

**Figure 8:**
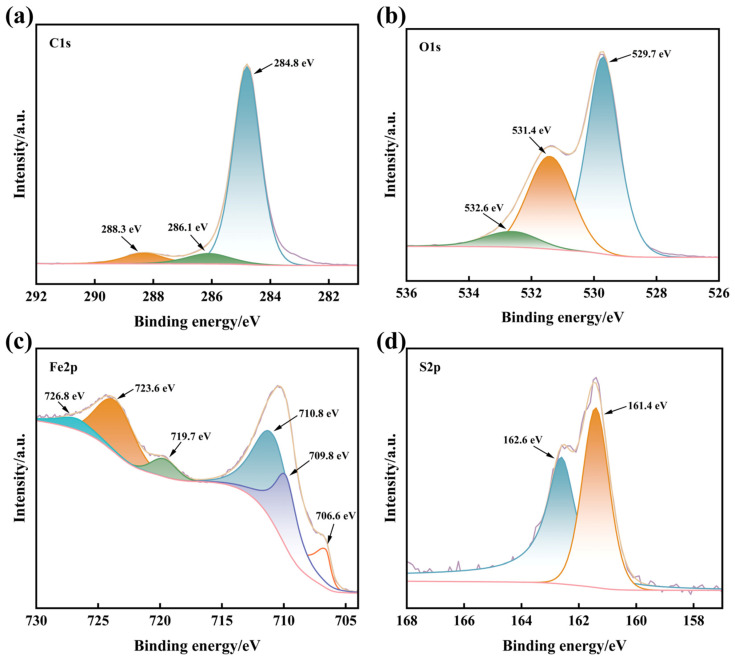
XPS analysis of disk wear scar surface after 1 wt% 16S lubrication: (**a**) C1s, (**b**) O1s, (**c**) Fe2p, (**d**) S2p.

In order to further characterize the anti-wear tribofilm mechanism of the two friction modifiers (16O and 16S), an XPS analysis was performed on the disk worn surface after lubrication by the studied oils, and the relevant results are shown in Figure 7 and Figure 8. It can be seen from the results that the spectra of C1s, O1s and Fe2p of the two friction modifiers are similar, and the binding energy peaks of C1s at 284.8 eV, 285.9 eV and 288.8 eV correspond to C-C, C-O and C=O, respectively [30,31]. The binding energy peaks in the O1s spectrum are 529.8 eV, 531.3 eV and 532.1 eV, respectively. The surface O element mainly exists in the chemical states of metal oxide, C-O and C=O [32]. The analysis results of Fe2p spectra show that the Fe element mainly exists in the form of FeO, Fe_2_O_3_ and Fe_3_O_4_ [33,34,35]. The binding energy peaks of the S element are 529.7 eV and 162.6 eV, indicating the formation of metal sulfides [36,37]. The above analysis results show that during the friction process, complex tribochemical reactions occur between the two friction modifiers and the metal friction pairs, and a new tribochemical reaction film containing C, O, Fe and S is formed, which effectively hinders the direct contact between the friction pairs, thus exhibiting higher anti-friction and anti-wear properties.

### 3.3. Adsorption Behavior Simulation

It can be seen from Table 2 that there are obvious differences in quantum chemical parameters between 16O and 16S. Compared with 16O, the EHOMO value of 16S is higher (closer to 0), indicating that its electron supply capacity is stronger, and it is easier to provide electrons to the metal surface during the friction process, thereby enhancing the interaction between the molecule and the metal surface. At the same time, the ELUMO value of 16S is lower, indicating that its ability to accept electrons is relatively stronger, so it has higher interfacial reactivity. The energy gap ΔE of 16S is only 0.182 Ha, which is lower than 0.247 Ha of 16O, indicating that its electronic transition requires lower energy, molecular chemical activity is higher, and electron transfer and interface adsorption behavior are more likely to occur. Correspondingly, the chemical hardness η of 16S is lower, while the softness S is higher, which further indicates that its molecular polarization ability is stronger, the structure is more active, and it is easier to form a stable adsorption film on the metal surface.

**Figure 9:**
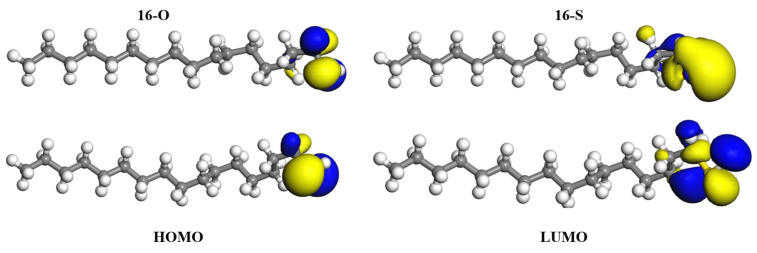
The HOMO and LUMO orbital distribution of 16O and 16S additives.

From Figure 9, it can be seen that the HOMO and LUMO orbitals of cetyl alcohol and cetyl mercaptan are mainly distributed near the polar functional groups at the end of the molecule, while the long-chain alkyl part has almost no obvious orbital distribution, indicating that the electronic activity of the molecule is mainly concentrated in the head group region. Among them, the HOMO orbital of the hexadecanol molecule is mainly concentrated near the hydroxyl group (-OH), indicating that there is a high electron cloud density around the oxygen atom, which is easy to provide electrons to the outside, so the hydroxyl group may be the main active site for its adsorption on the metal surface. The LUMO orbitals are also mainly distributed in the hydroxyl region, indicating that the intermolecular electron transfer behavior mainly occurs near the polar end groups. In contrast, the HOMO and LUMO orbitals of hexadecanethiol are more obviously concentrated near the thiol group (-SH); the orbital distribution is larger, and the electron cloud is more concentrated. This result indicates that the electronic activity of the sulfur atom is higher than that of the oxygen atom, and it has stronger electron supply and acceptance ability, so it is easier to interact with the metal surface and form a stable adsorption film. Due to the larger atomic radius and stronger polarization ability of sulfur atoms, stronger chemical adsorption is usually formed between sulfur atoms and metals. In general, the frontier orbitals of the two molecules are concentrated in the polar functional group region, but the orbital distribution of hexadecanethiol is more obvious, indicating that its interfacial reactivity may be stronger than that of hexadecanol, and it has greater potential advantages in the formation of a lubricating film on metal surfaces and interfacial adsorption.

**Figure 10:**
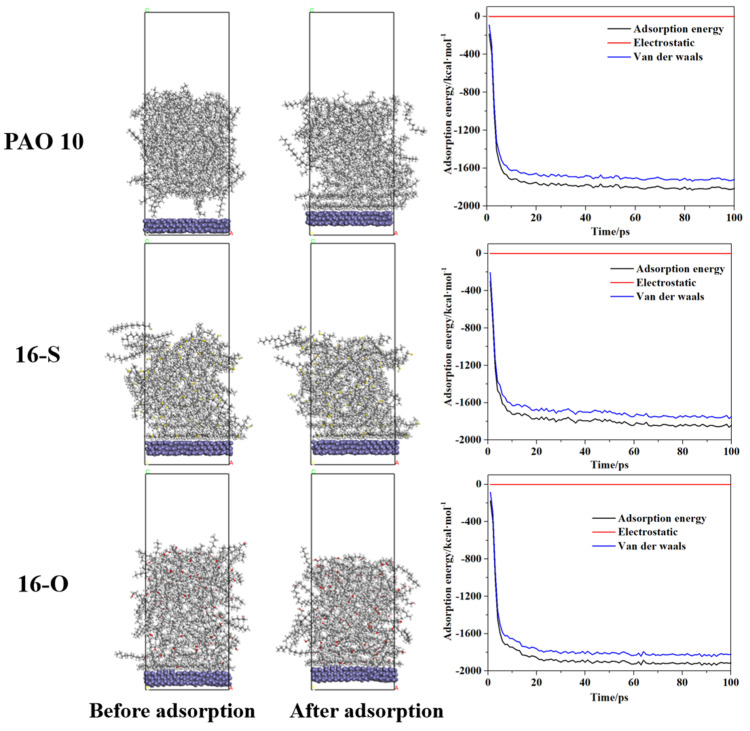
The adsorption configuration of PAO10, tested additives (16O and 16S), and their adsorption energy with the Fe surface.

Figure 10 shows the adsorption configuration changes in PAO10, 16S, and 16O molecules on the Fe surface and the corresponding adsorption energy evolution process. The results showed that the left and the middle are the molecular configurations before and after adsorption, respectively. The right side is the change curve of total adsorption energy, electrostatic interaction energy, and van der Waals interaction energy with time during the adsorption process. It can be seen from the adsorption configuration that the adsorption of PAO10 molecules on the Fe surface is relatively weak, and the molecules are mainly distributed in a loose state near the interface. After adsorption, no obvious dense adsorption layer is formed at the interface. In contrast, 16S and 16O molecules are obviously close to the Fe surface after adsorption, and a relatively stable adsorption layer is formed in the interface region. Among them, the adsorption of 16S molecules is more obvious, and some molecular chains are inclined or attached to the surface of Fe, indicating that there is a strong interfacial interaction between them and the metal surface.

It can be seen from the adsorption energy curve that the adsorption energy of the three systems decreased rapidly in the initial stage and gradually stabilized after about 10 ps, indicating that the molecules gradually completed the adsorption process on the Fe surface. On the whole, the absolute value of the adsorption energy of the 16-S system is the largest, followed by 16-O, and PAO 10 is the smallest, indicating that the adsorption capacity of hexadecanethiol and the Fe surface is the strongest, followed by hexadecanol, and PAO10 is relatively weak. In addition, the electrostatic interaction energy is generally small, while the van der Waals interaction energy makes the main contribution, indicating that the adsorption of the three molecules on the Fe surface is mainly dominated by the van der Waals interaction. At the same time, the 16S system showed a larger absolute value of adsorption energy, indicating that the presence of sulfur atoms enhanced the interfacial interaction between the molecule and the Fe surface, thereby improving the adsorption stability. This strong interfacial adsorption capacity is conducive to the formation of a stable boundary lubrication film, thereby improving the lubrication performance of the friction interface.

## 4. Conclusions

This study conducts a comparative analysis to evaluate the physicochemical and tribological properties of 1-hexadecanol (16O) and 1-hexadecanethiol (16S) as oil additives for lubricating steel tribosystems. The principal findings and insights are delineated below:

(1) The incorporation of the studied additives (16O and 16S) into PAO10 oil resulted in improved thermal stability and resistance to degradation by delaying the onset of oxidation. Notably, the results showed that the 16O addition had higher performance in its resistance to thermal degradation compared to the 16S additive.

(2) Tribological tests indicated that a concentration of 0.5 wt% is the most effective in reducing friction and wear. Compared to PAO10 base oil, the 16O and 16S additives reduced the friction coefficient and wear volume by 50–52% and 79.9–82.6%, respectively, under boundary lubrication conditions. The 16S addition was more effective in reducing wear, but the 16O additive was more effective in reducing friction.

(3) Morphological analysis of the friction scars demonstrated the effectiveness of the 16O and 16S additives in protecting surfaces from severe wear damage, resulting in smooth surfaces and similar surface protection performance for both additives. Furthermore, EDS analysis confirmed the deposition of the additive components within the tribolayer via tribochemical reactions, as shown by the XPS analysis.

(4) Adsorption behavior simulation revealed that the 16S additive had higher electron supply capacity, and it is easier to provide electrons to the metal surface compared with the 16O additive during the friction process, indicating that there is a strong interfacial interaction between them and the metal surface.

## Figures and Tables

**Table 1:** Kinematic viscosity, viscosity index and thermal decomposition temperature of tested oils.

Lubricant	Kinematic Viscosity/(mm^2^/s)		Viscosity Index	Thermogravimetric Temperature/°C		
	40 °C	100 °C		10%	20%	50%
PAO 10	64.275	10.359	149	252.92	278.32	315.62
0.3%-16O	64.732	10.363	148	314.11	338.80	379.80
0.5%-16O	64.040	10.206	146	317.07	342.91	382.14
1%-16O	62.324	10.118	149	310.74	337.18	377.57
0.3%-16S	65.433	10.340	146	272.74	295.94	334.74
0.5%-16S	62.828	10.214	150	284.41	312.21	353.61
1%-16S	63.144	10.650	160	270.73	295.73	335.73

**Table 2:** Quantum chemical parameters of 16O and 16S additives.

	EHOMO/Ha	ELUMO/Ha	ΔE/Ha	η/Ha	S/Ha-1
16O	−0.216	0.031	0.247	0.124	8.065
16S	−0.188	−0.006	0.182	0.091	10.989

## Data Availability

The original contributions presented in this study are included in the article. Further inquiries can be directed to the corresponding authors.
